# Structural Determinants of Alkyne Reactivity in Copper-Catalyzed Azide-Alkyne Cycloadditions

**DOI:** 10.3390/molecules21121697

**Published:** 2016-12-09

**Authors:** Xiaoguang Zhang, Peiye Liu, Lei Zhu

**Affiliations:** Department of Chemistry and Biochemistry, Florida State University, 95 Chieftan Way, Tallahassee, FL 32306-4390, USA; xizhang@chem.fsu.edu (X.Z.); peiyeliu@chem.fsu.edu (P.L.)

**Keywords:** alkyne, click chemistry, copper, reactivity, CuAAC

## Abstract

This work represents our initial effort in identifying azide/alkyne pairs for optimal reactivity in copper-catalyzed azide-alkyne cycloaddition (CuAAC) reactions. In previous works, we have identified chelating azides, in particular 2-picolyl azide, as “privileged” azide substrates with high CuAAC reactivity. In the current work, two types of alkynes are shown to undergo rapid CuAAC reactions under both copper(II)- (via an induction period) and copper(I)-catalyzed conditions. The first type of the alkynes bears relatively acidic ethynyl C-H bonds, while the second type contains an *N*-(triazolylmethyl)propargylic moiety that produces a self-accelerating effect. The rankings of reactivity under both copper(II)- and copper(I)-catalyzed conditions are provided. The observations on how other reaction parameters such as accelerating ligand, reducing agent, or identity of azide alter the relative reactivity of alkynes are described and, to the best of our ability, explained.

## 1. Introduction

The copper-catalyzed azide-alkyne cycloaddition (CuAAC) reaction [1,2] has been used as a principal molecular conjugation strategy by many in- and outside the chemistry community [3,4,5,6,7,8,9,10,11,12,13,14]. Identifying highly reactive azide and alkyne substrates of CuAAC carries the obvious benefit of increasing the efficiency of molecular conjugation. In bioconjugation applications, the additional benefit of applying reactive substrates is the reduced loading of copper catalyst that might be detrimental to cellular processes [15,16,17]. Our group reported the high reactivity of chelating azides in CuAAC [18,19,20,21]. These azides have subsequently been developed as bioconjugation tools applicable in living cells [22,23,24,25,26].

**Figure 1:**
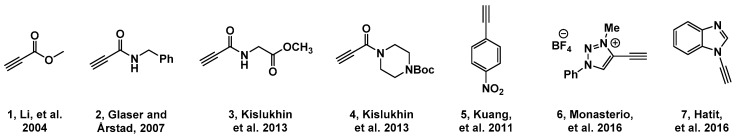
Reported fast-reacting alkynes in CuAAC.

Despite the emphasis placed upon copper acetylide in the mechanistic description of CuAAC [27,28], there have not been as many studies on the reactivity of terminal alkynes in CuAAC reactions. Isolated examples of reactive alkynes appeared soon after the discovery of CuAAC. Ju and coworkers found that methyl propiolate (**1**, Figure 1) has high reactivity in both thermal and copper-catalyzed cycloadditions with azides [29]. Propiolamide **2** was the most reactive in a ^18^F-radiolabeling study by Årstad and coworkers [30]. In the only systematic study on alkyne reactivity in CuAAC thus far, Finn and coworkers ranked the reactivity of thirteen alkynes under either bioconjugation or organic preparative conditions in the presence of an accelerating ligand [31]. Propiolamides (e.g., **3** and **4**) were found to be more reactive than other tested alkynes [31]. Our group found that the reactivity of *para*-substituted phenylacetylene increases as the substituent becomes more electron-withdrawing in copper(II) acetate-catalyzed CuAAC reaction in acetonitrile [20]. *p*-Nitroethynylbenzene (**5**) was the fastest-reacting alkyne in that regard [20].

Compounds **1**–**5** are alkynes bearing electron-withdrawing groups. Given the mechanistic revelations that the alkyne deprotonation to afford copper(I) acetylide is turnover limiting in CuAAC reactions [20,32], an electron-withdrawing group that would give a relatively low p*K*_a_ value to the C_sp_-H bond should enhance the alkyne reactivity. By this reasoning, the recently reported high CuAAC reactivity of alkyne **6** [33], in our opinion, could be attributed to its relatively high acidity. The same rationale can be applied to explain the reactivity of another newly reported alkyne **7** [34]. The lone-pair electrons on the C_sp_-attached nitrogen in **7** is delocalized in the benzimidazolyl group, which consequently acts as an electron-withdrawing group to increase the acidity of the C_sp_-H bond.

The purpose of the current work is to verify the high reactivity of relatively acidic terminal alkynes under both copper(II) and copper(I)-mediated CuAAC conditions, and to identify new classes of reactive alkynes. The azide reaction partner is chosen purposefully to be the chelating 2-picolyl azide. Therefore, the identified fast-reacting terminal alkynes may work together with 2-picolyl azide to afford a CuAAC substrate pair with optimal coupling efficiency.

## 2. Results and Discussion

### 2.1. Choice of Alkynes

**Figure 2:**
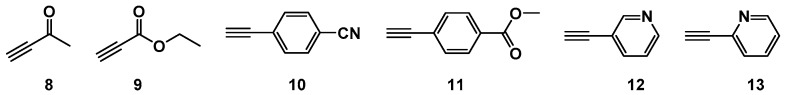
Alkynes bearing electron-withdrawing groups in addition to compounds **1**–**7**.

**Figure 3:**
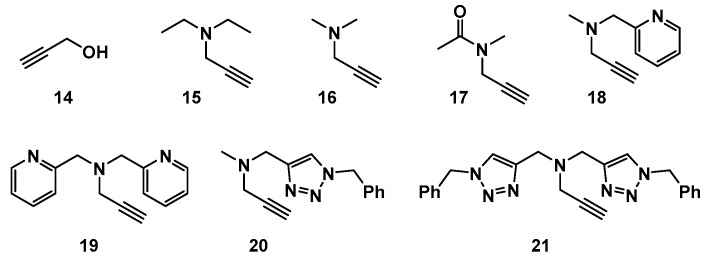
Alkynes with pendant coordination ligands.

**Figure 4:**
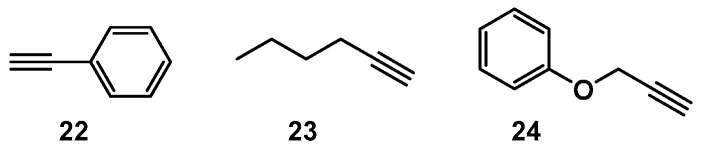
Alkynes that belong to neither group above.

The tested alkynes are separated in three categories: (a) electron-withdrawing group-bearing alkynes (Figure 2). These alkynes have relatively acidic C_sp_-H bonds. Therefore, deprotonation to afford acetylide, which is rate-determining in both alkyne oxidative homocoupling and CuAAC reactions, would be fast. The known examples of highly reactive alkynes in CuAAC (e.g., **1**–**7**) all fall into this category; (b) Alkynes with potentially copper-binding moieties (Figure 3). There have not been any studies on the reactivity of copper-binding alkynes. We are interested in this class of compounds because the alkyne themselves or their CuAAC products may be accelerating ligands. For example, the product between alkyne **21** and benzyl azide in a CuAAC reaction is tris[(1-benzyl-1*H*-1,2,3-triazol-4-yl)methyl]amine (TBTA), which is a known ligand for accelerating CuAAC [35]; (c) Alkynes that belong to neither group above (Figure 4).

### 2.2. The ^1^H-NMR Assay

**Scheme 1:**
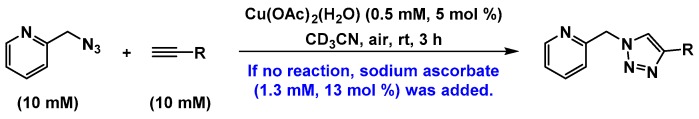
Reaction used in the ^1^H-NMR time course assay to evaluate alkynes. The actual concentrations of reaction ingredients are in parentheses.

The reactivity of alkynes was compared in a copper(II) acetate (5 mol % of the limiting reagent alkyne) catalyzed reaction [18,19] with the chelating 2-picolyl azide (Scheme 1). The reactive 2-picolyl azide was selected so that the concentrations of substrates can be lowered to NMR-manageable values (i.e., 10 mM or lower) for online continuous monitoring [20]. If no product formed after 3 h of reaction time, sodium ascorbate (13 mol %) was added to completely reduce copper(II) to copper(I), which in most cases kick-started the reaction.

**Figure 5:**
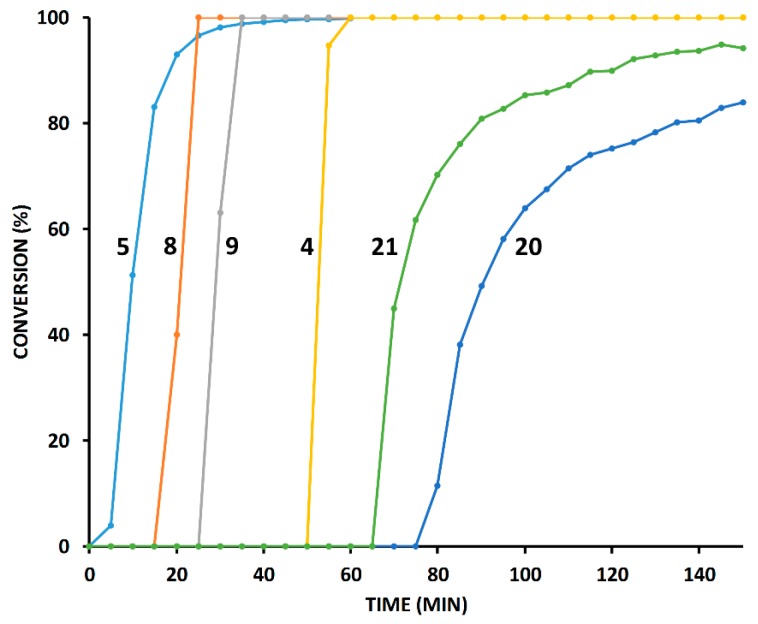
Conversion values of the limiting reagent alkyne over time before the 3-h mark as acquired via continuous monitoring by ^1^H-NMR at r.t. The spectra were taken every 5 min. Alkyne (10 mM), 2-picolyl azide (10 mM), Cu(OAc)_2_·H_2_O (0.5 mM).

Among all the alkynes tested, six of them achieved significant conversion within the first 3 h without the addition of sodium ascorbate (Figure 5). *p*-Nitrophenylacetylene (**5**) has the shortest induction period, followed by that of ethynyl methyl ketone (**8**), ethyl propiolate (**9**), and propiolamide **4**. Alkynes **20** and **21** that contain the copper-binding triazolylmethyl group have longer induction periods. The length of the induction and the triazole formation efficiency appear to followed the acidity of the ethynyl protons. The propiolyl derivatives **4**, **8**, **9** have the highest triazole formation efficiency—the reactions are completed within 5–10 min after the induction. This type of alkynes however is susceptible to conjugate nucleophilic addition at the terminal carbon. Alkynes **20** and **21** are precursors of CuAAC-accelerating ligands. They may also be self-accelerating. The relatively high CuAAC reactivity of **20** and **21** with no conjugate addition reactivity makes them attractive substrate manifolds to build reactive and selective conjugation linkers in complexed environments containing various nucleophilic species.

**Figure 6:**
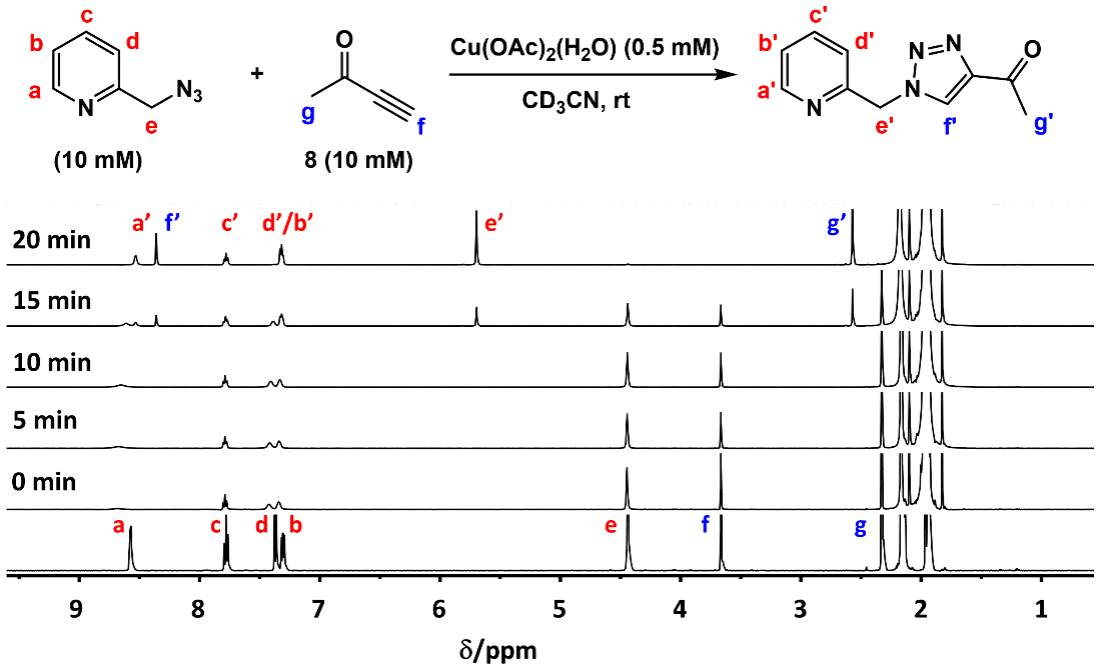
The evolution of ^1^H-NMR of the reaction between 2-picolyl azide and ethynyl methyl ketone (**8**). Cu(OAc)_2_·H_2_O was added at ‘0 min’.

Representative ^1^H-NMR data from these reactions are discussed herein. Some information on how azide and/or alkyne interacted with the copper catalyst or pre-catalyst was obtained from these data. The spectra shown in Figure 6 were acquired during the reaction between 2-picolyl azide and ethynyl methyl ketone **8**. The mixture of azide and alkyne substrates in CD_3_CN in the absence of the copper catalyst gave the spectrum at the bottom. Upon addition of Cu(OAc)_2_·H_2_O at 5 mol % of the limiting reagent alkyne, the pyridyl protons that are *ortho* (proton a) and *meta* (protons b and d) to nitrogen were broadened (spectrum ‘0 min’), suggesting coordination of copper at the pyridyl nitrogen of 2-picolyl azide. Line-broadening was not observed for the alkyne proton peaks (protons g and f). Therefore, in this case when the azide is chelating, copper (pre)catalyst is bound with the azide substrate at the outset of the reaction, before the formation of copper acetylide.

**Figure 7:**
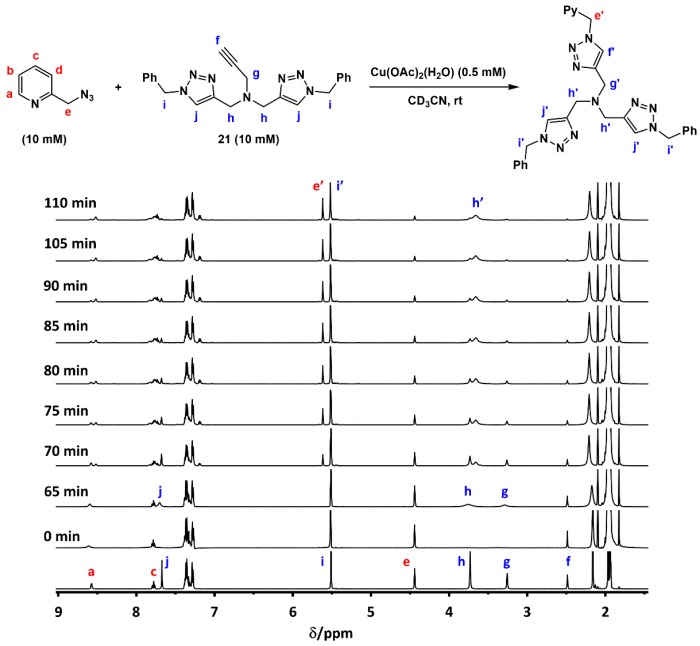
The evolution of ^1^H-NMR of the reaction between 2-picolyl azide and alkyne **21**. Cu(OAc)_2_·H_2_O was added at ‘0 min’. Py = 2-pyridyl.

The ^1^H-NMR data of the reaction between 2-picolyl azide and alkyne **21** are shown in Figure 7. In this case, after the addition of Cu(OAc)_2_·H_2_O (spectrum ‘0 min’), the signals of protons g, h, and j on **21** completely disappeared (i.e., broadened down to the baseline), indicative of binding of the alkyne to copper(II) at the outset of the reaction. Therefore, the high reactivity of alkyne **21**, which shall have a relatively high p*K*_a_ value in the realm of aliphatic alkynes, can be at least partially attributed to its ability to recruit copper, even in the pre-catalyst form. As the spectrum transitioned during the induction period from ‘0 min’ to ’65 min’, the peaks of protons g, h, and j reappeared as broad singlets. The reappearances of these protons on the alkyne as the triazole formation is imminent suggest that the (partial) reduction of paramagnetic copper(II) to diamagnetic copper(I) is prerequisite to the triazole formation.

**Figure 8:**
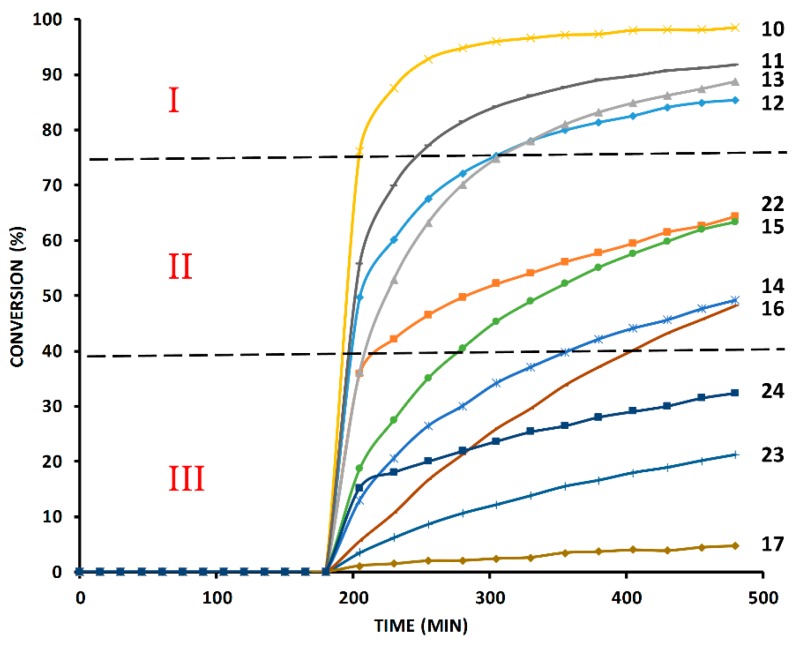
Conversion values over time beyond the 3-h mark at which sodium ascorbate (1.3 mM, 13 mol %) was added as acquired via continuous monitoring by ^1^H-NMR at r.t. Alkyne (10 mM), 2-picolyl azide (10 mM), Cu(OAc)_2_·H_2_O (0.5 mM, 5 mol %). Sodium ascorbate (1.3 mM) was added at the 3-h mark.

Triazole production was not observed with other alkynes during the first 3 h under the conditions listed in the caption of Figure 5 [36]. For these reactions, at the 3-h mark, sodium ascorbate (13 mol % of the limit reagent alkyne) was delivered to the reaction mixture in the NMR tube, and the reaction was monitored for an additional 5 h. Most alkynes except two (**18** and **19** in Figure 3) started conversion to triazole immediately after the addition of sodium ascorbate and achieved various levels of completion after 5 h. The alkynes are divided into three groups in Figure 8 based on reactivity. Group I consists of alkynes **10**–**13**, all ethynyl arenes with electron-withdrawing substituents, which had achieved over 80% conversion. Phenylacetylene (**22**), propargylic alcohol (**14**) and amines **15**, **16** constituted Group II, while the aliphatic alkynes **23**, **24**, and the acylated propargyl amine **17** have the lowest conversion values and therefore are in Group III. The 2-picolyl-functionalized propargyl amines **18** and **19** failed to react.

**Scheme 2:**
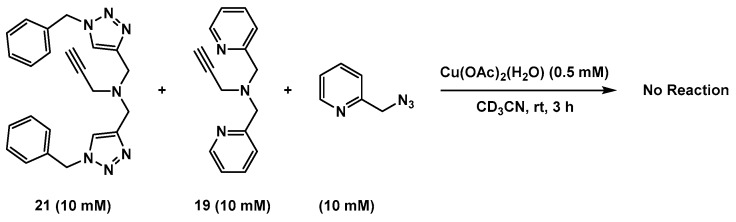
The effect of alkyne **19** on the reaction between alkyne **21** and 2-picolyl azide.

We attribute the low reactivity of alkynes **18** and **19** to their abilities in forming strong complexes with copper(II or I), which reduce the catalytic potency of the metal. Compound **21** is an analog of compound **19**; yet **21** is reactive without the participation of sodium ascorbate (Figure 5). The addition of an equal molar amount of alkyne **19** in the reaction mixture of **21** and 2-picolyl azide resulted in no reaction (Scheme 2), which is consistent with the argument that **19** binds copper stronger than **21**, and consequently it hijacks copper from participating in the reaction between **21** and 2-picolyl azide.

From the data in Figure 5 and Figure 8, it can be concluded that: (a) electron-deficient alkynes such as ethynyl ketones, propiolates and propiolamides, and electron-withdrawing group-substituted ethynyl arenes are reactive in both copper(II)- and copper(I)-catalyzed conditions; (b) *N*-triazolylmethyl-substituted propargyl amines are also fast reacting. Those compounds might act as accelerating ligands for their own conversion to triazoles; (c) Inclusion of amino or hydroxyl groups in an aliphatic alkyne adjacent to the ethynyl group increases its CuAAC reactivity; and (d) the presence of a multidentate ligand of a strong copper affinity, such as di(2-picolyl)amino [37], slows the reaction down. It was tempting to call the *N*-triazolylmethyl-substituted propargyl amines **20** and **21** “chelating alkynes” in the same manner as “chelating azides”, which would be suggestive of high reactivity. However, when a stronger copper ligand is used in place of a triazolylmethyl group such as in those **18** and **19**, the reactivity drops precipitously. Therefore, we do not advocate the use of the term “chelating alkynes”, which would likely give a false expectation of high reactivity.

### 2.3. Reaction with the Non-Chelating Benzyl Azide

**Figure 9:**
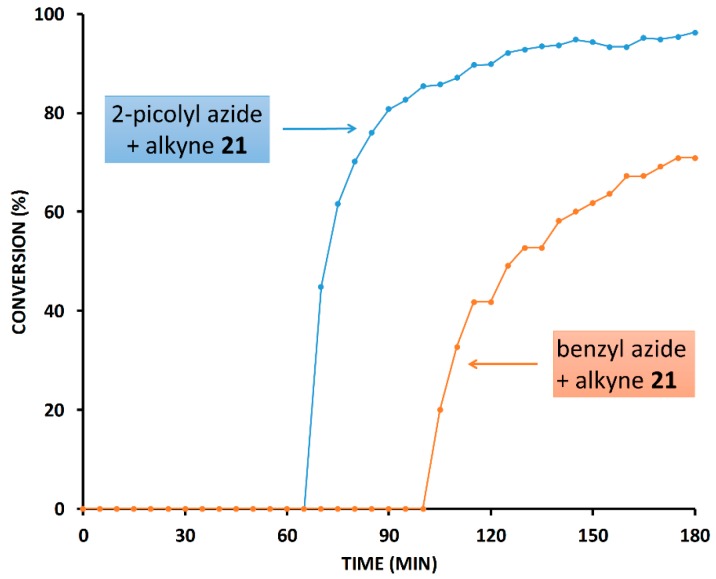
Conversion values of alkyne **21** over time as acquired via continuous monitoring by ^1^H-NMR at r.t. The spectra were taken every 5 min. alkyne **21** (10 mM), 2-picolyl azide or benzyl azide (10 mM), Cu(OAc)_2_·H_2_O (0.5 mM).

For practical reasons, we focus mostly on the six fast-reacting alkynes shown in Figure 5 in the following subsections. We would like to know whether they would also engage azides without chelation activation, such as benzyl azide, under the conditions depicted in Figure 5. Different from the reactions with 2-picolyl azide, no reaction occurred between propiolamide **4** or ethynyl methyl ketone **8** and benzyl azide within 3 h, while alkyne **21**, which is the second slowest among the “lead pack” in Figure 5, started conversion to triazole at 100-min mark (orange trace in Figure 9). 2-Picolyl azide, on the contrary, initiated the reaction at 65-min mark under the same conditions (Figure 9, blue, reproduced from Figure 5). From these data, it can be concluded that (a) the nature of the azide has an overriding effect on the rate of CuAAC, as the chelating 2-picolyl azide reacts faster than benzyl azide regardless of the identity of alkyne; (b) the reactivity rank of alkyne depends on the nature of azide—reactivity reversion was observed for alkynes **4**/**8** and **21** when they pair up with 2-picolyl azide or benzyl azide.

### 2.4. Comparison of Fast-Reacting Alkynes with a Normalized Amount of Copper(I)

**Figure 10:**
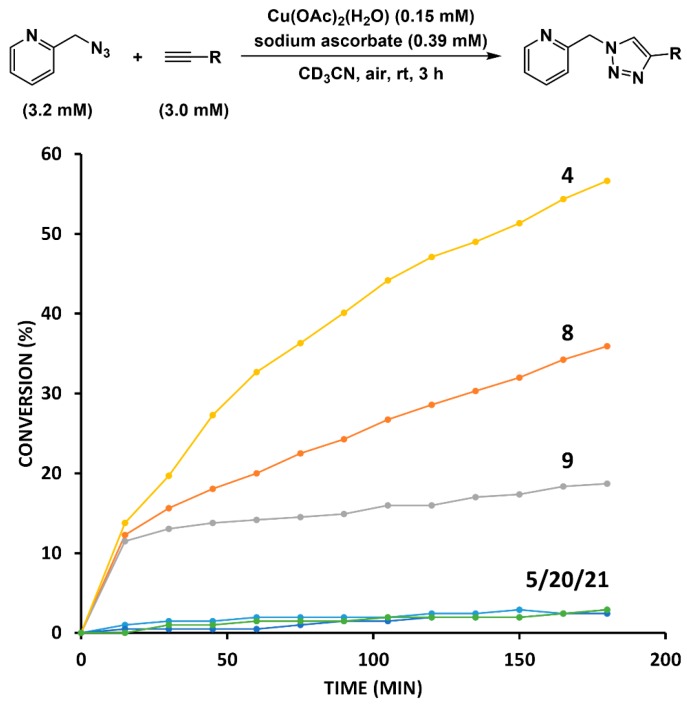
Conversion values of alkynes **4**, **5**, **8**, **9**, **20**, and **21** over time as acquired via continuous monitoring by ^1^H-NMR at r.t. The spectra were taken every 5 min. Concentrations: alkyne (3.0 mM), 2-picolyl azide (3.2 mM), Cu(OAc)_2_·H_2_O (0.15 mM), and sodium ascorbate (0.39 mM).

As shown in Figure 5, the triazole formation phases for four of the alkynes (compounds **4**, **5**, **8**, **9**) are exceptionally fast, being complete within 5–10 min after the induction period. Because there is no information on how much copper(I) catalyst was produced during the induction periods of these reactions, one shall not attribute the differences in reaction rates of these four compounds solely to the differences in their inherent reactivity. For comparing their reactivity, another set of experiments with lower substrate concentrations and in the presence of an excess amount of sodium ascorbate relative to copper to normalize the amount of copper(I) catalyst were conducted (Figure 10).

The reactions were run at lower concentrations than those employed in the last subsection. The limiting reagent alkyne was set at 3.0 mM instead of 10 mM. As expected, the reactions were slower so that the differences between the reactivity of these alkynes can be unambiguously observed. The induction periods were eliminated in the presence of sodium ascorbate, and the propiolyl type alkynes **4**, **8**, and **9** reacted faster than **5**, **20**, and **21** (Figure 10). Among the propiolyls, propiolamide **4** has the highest reactivity, followed by ethynyl ketone **8** and propiolate **9**.

### 2.5. Reduction of Copper(II) Acetate by Ascorbate Alters the Pathway

**Figure 11:**
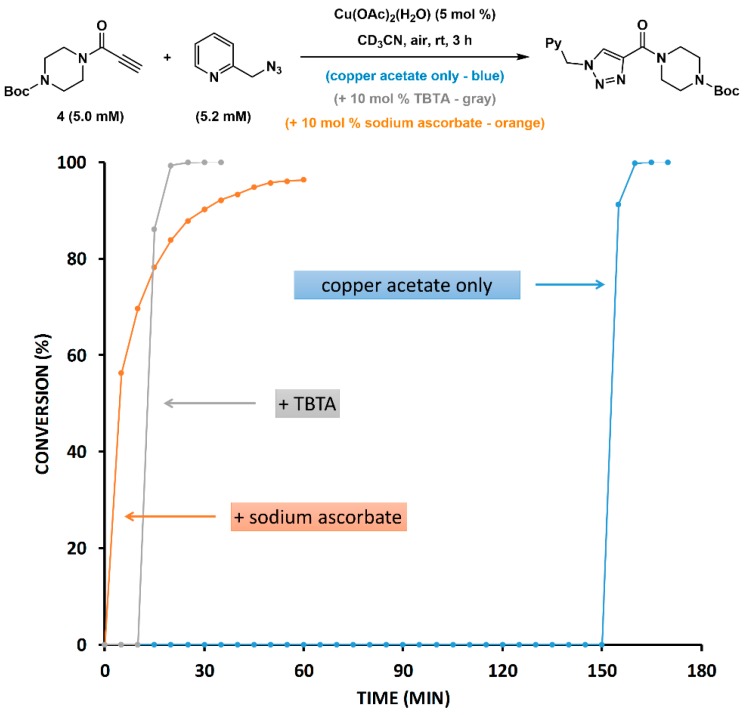
The kinetic traces of the reaction between 2-picolyl azide and alkyne **4** to show the effect of sodium ascorbate (orange) and TBTA (gray). Py = 2-pyridyl.

Although the addition of sodium ascorbate eliminated the induction period, it might also have altered the structure of the active catalyst formed under the non-reductive conditions. The reaction between propiolamide **4** and 2-picolyl azide was used to examine this possibility. After a long induction period in the reaction without sodium ascorbate, the product formation is steeper (i.e., faster) than the reaction with copper(II) fully reduced by sodium ascorbate at the outset (Figure 11). Considering that only a (small) fraction of copper was reduced to copper(I) in the former case, the activity of the catalyst formed after the induction reaction was extraordinary. Structural identification of that catalyst, likely a 2-picolyl azide coordinated dinuclear copper(I) acetate [20], is a target of future study.

### 2.6. The Effect of TBTA on the Slow Alkyne Substrates

**Figure 12:**
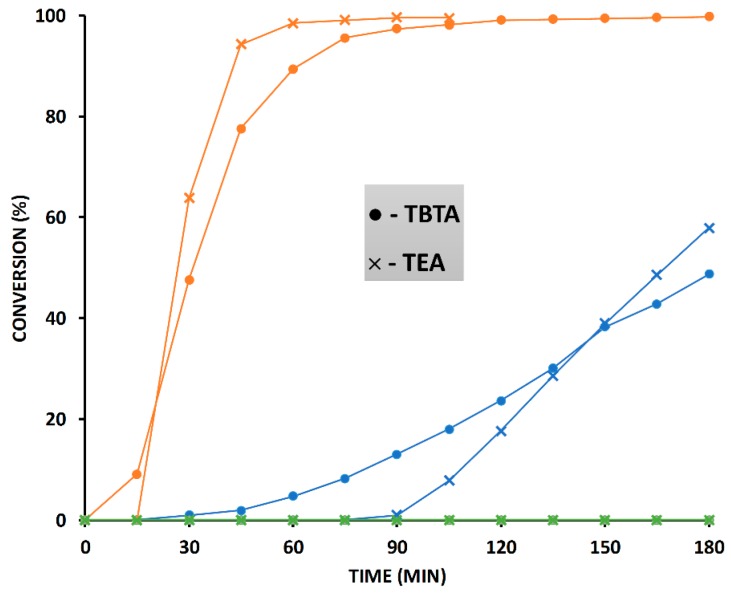
The kinetic traces of reactions between 2-picolyl azide and alkynes **10** (orange), **22** (blue), and **23** (green) in the presence of TBTA (filled circles) or TEA (crosses).

TBTA [35] is a ligand that is known to accelerate the triazolyl ring formation step in CuAAC. Among the alkynes listed in Figure 8, which are those that did *not* react within the first 3 h, three alkynes (**10**, **22**, **23**) were selected from each group to be subjected under copper(II) acetate only conditions in the presence of TBTA (10 mol %) [19,38]. TBTA was shown to enhance the reactivity of the relatively acidic *p*-ethynylbenzonitrile (**10**) and phenylacetylene (**22**) (Figure 12). TBTA therefore likely shortens the induction period by accelerating the alkyne oxidative homocoupling reaction. This function of TBTA was also shown in the reaction involving the more reactive alkyne **4** at a lower substrate concentration (Figure 11). No reaction occurred to 1-hexyne **23**, the alkyne in the lowest reactivity category (Group III in Figure 8), under the same conditions.

**Figure 13:**
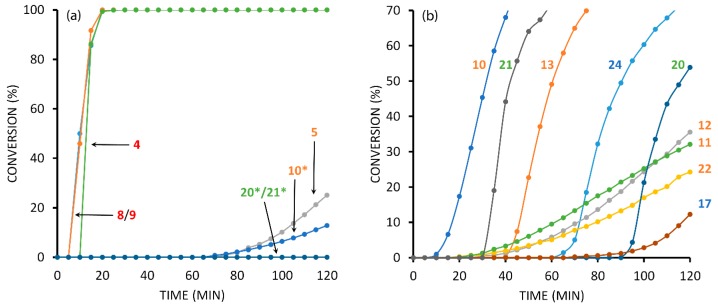
(**a**) Triazole formation over time of alkynes **4**, **5**, **8**, **9**, **10**, **20**, and **21** (5 mM each) with 2-picolyl azide (5 mM) in the presence of Cu(OAc)_2_(H_2_O) (0.25 mM) and TBTA (0.5 mM) in CD_3_CN at r.t. for 2 h. *: substrates reexamined at a higher concentration; (**b**) triazole formation over time of alkynes **10**–**13**, **17**, **20**–**22**, and **24** (10 mM each) with 2-picolyl azide (10 mM) in the presence of Cu(OAc)_2_(H_2_O) (0.5 mM) and TBTA (1.0 mM) in CD_3_CN at rt for 2 h. Compound numbers in red: propiolyl derivatives; orange: ethynyl aromatics; blue: ethynyl aliphatics; and green: multidentate copper-binding ligands.

For the sake of completeness, the reactivity of all alkynes **1**–**24** were compared in the presence of 10 mol % TBTA. For the highly reactive alkynes **4**, **5**, **8**, **9**, **20**, and **21** listed in Figure 5, the concentrations of the substrates were cut to 5 mM. Propiolyl (HC≡C-(C=O)-) derivatives **4**, **8**, and **9** maintained their superior reactivity, while *p*-nitrophenylacetylene (**8**) was falling far behind, and *N*-triazolylmethyl-substituted propargyl amines **20** and **21** failed to produce triazole in 2 h (Figure 13a). Alkynes **20** and **21** were reexamined with the rest of alkynes at the original substrate concentrations (10 mM each for alkyne and 2-picolyl azide) in the presence of 10 mol % TBTA. The reactivity of these alkynes in broad strokes matches the conclusions from the ligand-free experiments (Figure 5 and Figure 8)—ethynyl arenes are more reactive than aliphatic terminal alkynes (Figure 13b). Within ethynyl arenes, electron-withdrawing substituents increase the reactivity. The *N*-triazolylmethyl-substituted propargyl amines **20** and **21** still show rapid triazole-forming phases, with relatively long induction periods. The pyridyl analogs of both compounds—**18** and **19**—failed to react under the same conditions. Several alkynes jumped up on the order of reactivity with the assistance of TBTA. 2-Ethynylpyridine (**13**) was identified for its relatively high reactivity; this compound was reported as an accelerating additive for CuAAC reactions [39]. Alkyne **24** was another one that showed surprising reactivity in the presence of 10 mol % TBTA. This compound also exhibited high reactivity in the study by Finn and coworkers [31]. The acylated propargyl amine **17** saw the uptick of its reactivity aided by TBTA. We do not have a model to relate the structures of these alkynes to the observations on their TBTA-dependent reactivity. The rest of the alkynes not listed in Figure 13 did not react under the given conditions.

**Figure 14:**
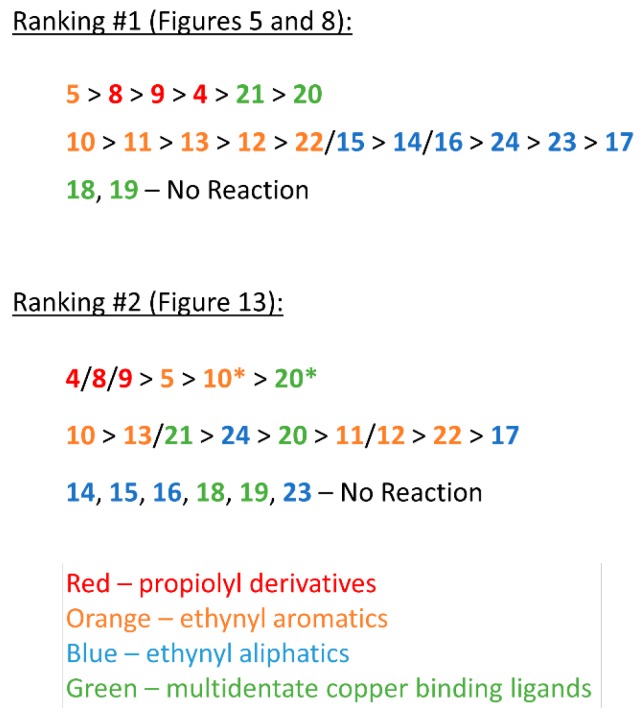
Summary of reactivity rankings of alkyne **1**–**24** in CuAAC determined in Figure 5, Figure 8 and Figure 13.

The rank of reactivity of alkyne **1**–**24** are summarized in Figure 14. Again, propiolyl derivatives are the most reactive, followed by ethynyl arenes that are activated by electron-withdrawing substituents. Aliphatic alkynes have relatively low reactivity. Copper-binding neighboring groups may elevate (e.g., **20**, **21**) or attenuate (e.g., **18**, **19**) the reactivity of terminal alkynes. The structure-function relationship of these alkynes is a topic of ongoing studies.

What is the mechanistic basis for the accelerating effect of TBTA, and other poly(triazolyl) ligands, on CuAAC reactions that start with either copper(I) or copper(II) precatalyst? It has been hypothesized that TBTA stabilizes copper in the active +1 oxidation state [35,40]. However, in our opinion, which we presented in a review article [41], there is no definitive evidence to fully establish this copper(I)-stabilizing ability. From the data of TBTA-accelerated reactions in this work, TBTA likely accelerates the inducting alkyne oxidative homocoupling reaction. This argument was substantiated as TBTA showed accelerating effect on the oxidative homocoupling of phenyacetylene (Table 1). Under the given conditions, 57% (based on ^1^H-NMR, entry 1) of phenylacetylene was converted to diyne in the presence of TBTA (10 mol %), while without TBTA no oxidative reaction occurred (entry 2). The accelerating effect of TBTA on the alkyne oxidative homocoupling reaction (the Glaser reaction) was also reported in a previous work of our group [38]. 2-Picoline (entry 3), which resembles 2-picolyl azide, showed a more modest accelerating effect, while triethylamine (TEA) did not (entry 4).

Being a tertiary amine, TBTA is also a base. We therefore used triethylamine (TEA) to model the basicity of TBTA to gauge how much the basicity of TBTA contributes to the accelerating effect on CuAAC. When TEA was used in place of TBTA, the reactions with both alkynes **10** and **22** were accelerated. For both alkynes, TEA-involved reactions experienced longer induction periods yet faster triazole production than those with TBTA participation, which means that TEA is not as effective as TBTA in catalyzing the Glaser reaction (alkyne oxidative homocoupling) but may form a more potent copper(I) catalyst of the CuAAC reaction.

**Scheme 3:**
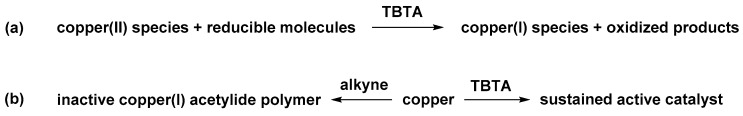
Possible functions of TBTA in CuAAC.

Based on the comparison of the effects of TBTA and TEA on the copper(II) acetate-mediated cycloaddition, it is possible that one of the functions of TBTA is the kinetic activation of copper(II) for oxidative chemistry, which re-engages oxidized copper catalyst in CuAAC reaction (Scheme 3a). This argument differs from the thermodynamic model that TBTA stabilizes the +1 oxidation state of copper. TBTA as a ligand might also increase the longevity of the copper catalyst (e.g., by reducing the likelihood of copper(I) acetylide polymer formation, Scheme 3b). However, the TBTA/copper complex might *not* be the most reactive copper(I) catalytic species in these reactions. As shown in Figure 12, the copper catalyst produced in the TEA-involved reactions was more potent. In Figure 11, the copper catalyst produced from ligand-free induction period (blue trace) is at least as reactive as the one with the participation of TBTA (gray trace).

### 2.7. Competition Experiments

**Figure 15:**
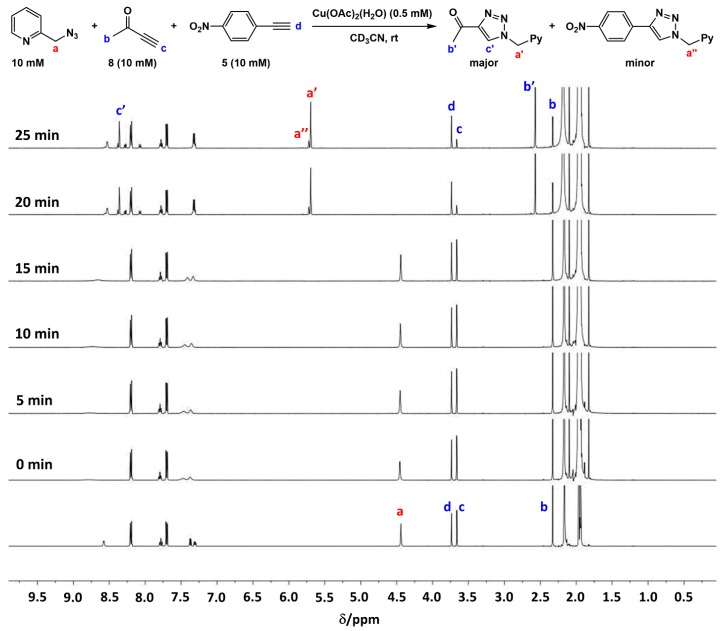
The evolution of the ^1^H-NMR spectra of the reaction between alkynes **5** (10 mM), **8** (10 mM), and 2-picolyl azide (10 mM). Cu(OAc)_2_·H_2_O (0.5 mM) was added in (‘0 min’). Py = 2-pyridyl.

Several combinations of the alkynes (**5**, **8**, **10**, **21**) were used to react with 2-picolyl azide. The purposes of these competition experiments are to (a) verify the reactivity ranking that was established using the individual NMR experiments; and (b) observe how they may interfere with each other’s reaction pathway. When *p*-nitrophenylacetylene (**5**), ethynyl methyl ketone (**8**) and 2-picolyl azide were mixed at 10 mM each, after an induction period of 20 min, the reaction proceeded rapidly that involved primarily alkyne **8** (Figure 15). Comparing to the reactivity ranking and the length of induction periods acquired from the individual experiments, which had alkyne **5** more reactive than alkyne **8** without sodium ascorbate (Figure 5), the observation from the competition experiment suggests that alkyne **8** interacts with copper(II) acetate precatalyst stronger than alkyne **5**, which lengthens the induction period. Yet alkyne **8** is more reactive in the post-induction, triazole-production step, as it outcompetes alkyne **5** (Figure 15).

**Figure 16:**
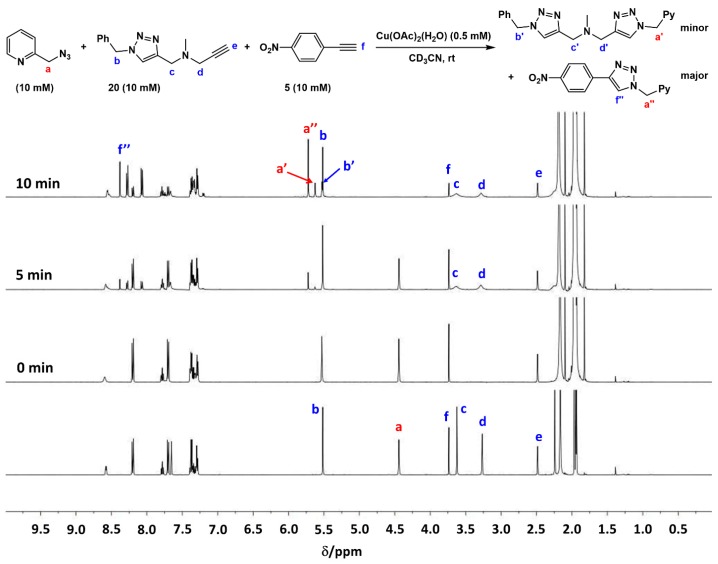
The evolution of the ^1^H-NMR spectra of the reaction between alkynes **5** (10 mM), **20** (10 mM), and 2-picolyl azide (10 mM). Cu(OAc)_2_·H_2_O (0.5 mM) was added in (‘0 min’). Py = 2-pyridyl.

Mixing alkynes **5**, **20**, and 2-picolyl azide at 10 mM each replicated the short induction period (5 min) that was observed with alkyne **5** alone (Figure 16). Compound **5** was consumed faster than compound **20**, therefore confirming the higher reactivity of alkyne **5** than that of **20**. The reaction between alkyne **20** and 2-picolyl azide was accelerated by the presence of alkyne **5**, because the relatively fast homocoupling of alkyne **5** provided copper(I) that was shared with alkyne **20**, effectively cutting its induction period from previously measured 80 min to 5 min.

**Figure 17:**
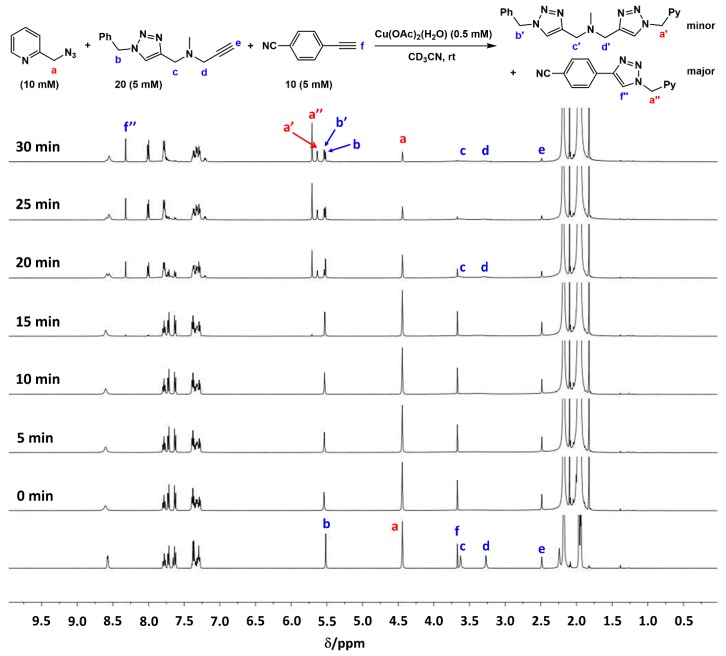
The evolution of the ^1^H-NMR spectra of the reaction between alkynes **10** (5 mM), **20** (5 mM), and 2-picolyl azide (10 mM). Cu(OAc)_2_·H_2_O (0.5 mM) was added in (‘0 min’). Py = 2-pyridyl.

In the last competition experiment, alkyne **20**, which reacted with 2-picolyl azide within the first 3 h (Figure 5), and alkyne **10**, which did not until sodium ascorbate was added at 3-h mark (Figure 8), were mixed at 5 mM each with 2-picolyl azide (10 mM). Interestingly, after a relatively short induction period of 20 min (alkyne **20** alone at 10 mM experienced an 80-min induction period), the triazole formation of *both* alkynes started (Figure 17). Alkyne **10**, which by itself was unreactive during the first 3 h under otherwise same conditions, reacted *faster* than alkyne **20** when they were mixed together. Presumably, alkyne **20** acted as a ligand for copper to accelerate the homocoupling induction reaction of alkyne **10**. As soon as copper(I) is produced via the induction reaction, the more acidic alkyne **10** dominates the triazole formation phase.

## 3. Experimental Section

### 3.1. Materials and General Methods

Reagents and solvents were purchased from various commercial sources and used without further purification unless otherwise stated. Analytical thin layer chromatography (TLC) was performed using precoated TLC plates with silica gel 60 F254. Flash column chromatography was performed using 40–63 μm (230–400 mesh ASTM) silica gel as the stationary phase. ^1^H- and ^13^C-NMR spectra were recorded at 500 and 125 MHz respectively, at 295 K unless otherwise noted. The chemical shifts (δ) were recorded in ppm relative to the residual CHCl_3_ or CHD_2_CN as internal standards. High resolution mass spectra (HRMS) were obtained under electrospray ionization (ESI) using a time-of-flight (TOF) analyzer. Alkynes **4** [31], **17** [42], **18** [43], and **19** [44] were prepared using reported procedures. Other alkynes were acquired from commercial sources. For ensuring reproducibility, alkynes **8**, **14**–**16**, and **22**–**24** were re-purified via distillation, while alkynes **5**, **10**, **11**, and **13** were re-purified via silica column chromatography.

### 3.2. Synthesis of Compound ***20*** (Scheme 4)

In a round-bottom flask compound **25** [44] (303 mg, 1.2 mmol) was dissolved in THF (4.8 mL). K_2_CO_3_ (3.2 mmol, 442 mg) and *N*-methyl propargylamine (84 µL, 1.0 mmol) were added. The flask was equipped with an argon balloon and the reaction was let go at r.t. for 24 h. The reaction mixture was then diluted with ethyl acetate, and passed through a pad of K_2_CO_3_. The solvent was removed under reduced pressure to afford the product as a yellow oil (144 mg, 60% yield). FTIR (neat, ν_max_ (cm^−1^)): 3293 (C≡C-H), 2104 (C≡C); ^1^H-NMR (CDCl_3_) δ/ppm 7.41 (s, 1H), 7.39–7.33 (m, 3H), 7.27–7.25 (m, 2H), 5.51 (s, 1H), 3.71 (s, 2H), 3.31 (d, *J* = 2.4 Hz, 2H), 2.33 (s, 3H), 2.25 (t, *J* = 2.5 Hz, 1H); ^13^C-NMR (CDCl_3_) δ/ppm 145.4, 134.7, 129.2,128.9, 128.2, 122.7, 78.5, 73.7, 54.3, 50.7, 45.3, 41.7; HRMS(ESI): *m*/*z* [M + Na^+^] calcd for C_14_H_16_N_4_Na, 263.1273; found, 263.1269.

### 3.3. Synthesis of Compound ***21*** (Scheme 5)

In a round-bottom flask compound **26** [45] (359 mg, 1.0 mmol) was dissolved in THF (5 mL). K_2_CO_3_ (520 mg, 3.8 mmol) and propargyl bromide (131 µL, 1.2 mmol, in 80 wt % toluene solution) were added. The flask was equipped with an argon balloon. The reaction was run at r.t. for 24 h, followed by dilution with EtOAc (50 mL), and was passed through a pad of K_2_CO_3_. Solvent was removed under reduced pressure to afford a yellow oil crude product, which was purified by recrystallization (CH_2_Cl_2_/diethyl ether) to a white solid (167 mg, 42% yield). ^1^H-NMR (CDCl_3_): δ/ppm 7.48 (s, 2H), 7.38–7.33 (m, 6H), 7.26–7.25 (m, 2H), 5.50 (s, 4H), 3.81 (s, 4H), 3.32 (d, *J* = 2.4 Hz, 2H), 2.23 (t, *J* = 2.5 Hz, 1H). This compound was reported [46].

### 3.4. General Procedure for ^1^H-NMR Reaction Monitoring Experiments

The stock solutions were prepared in CD_3_CN for all reaction components except Cu(OAc)_2_·H_2_O and sodium ascorbate. The Cu(OAc)_2_·H_2_O stock solution was prepared in CH_3_CN. This solution needs to be freshly made before the ^1^H-NMR monitoring experiments to ensure reproducibility. Sodium ascorbate stock solution was prepared in deionized water. CD_3_CN (372.5 μL), alkyne (50 μL, 100 mM), and 2-picolyl azide or benzyl azide (52.5 μL, 100 mM) were added sequentially into an NMR tube and mixed by flipping the sealed NMR tube 6 times. A ^1^H-NMR spectrum of the mixture was taken. Afterwards, Cu(OAc)_2_·H_2_O (25 μL, 10 mM) was added into the NMR tube and the sealed tube was flipped 6 times to obtain a homogenous solution. The sample was inserted and the reaction was monitored under a multiple scan mode for 3 h. The interval of the scans was 5 min. If there was no reaction after 3 h, sodium ascorbate (3.2 μL, 200 mM) was added into the NMR tube and mixed in the same way to generate Cu(I) in situ. The reaction was followed by ^1^H-NMR under the same multiple scan mode for another 5 h.

For other experiments, in addition to adjusting the concentrations of the abovementioned reaction components (azide, alkyne, Cu(OAc)_2_·H_2_O, and sodium ascorbate), appropriate amounts of the stock solutions of TBTA or TEA (both in CD_3_CN) were added as required.

All the concentrations listed in the procedure are stock solution concentrations of individual reaction participants. The final concentrations of reaction components are listed in the figures and figure captions.

### 3.5. Oxidative Homocoupling of Phenylacetylene (Table 1)

To a 10-mL round-bottom flask *t*-BuOH (1 mL) was added. Phenylacetylene (46.1 µL, 0.42 mmol), additive (0.02 mmol), Cu(OAc)_2_·H_2_O (40 mg, 0.2 mmol) were added in this order. The reaction mixture was stirred under air for 18 h. After the reaction finished, the reaction mixture was diluted with ethyl acetate (50 mL), then washed with saturated brine three times. The organic layer was dried over Na_2_SO_4_, and the solvent was removed under reduced pressure. The ^1^H-NMR spectrum of the residue was taken, from which the conversion to diyne was calculated from the analysis of the 7.4–7.6 ppm region (underlined). Phenylacetylene: ^1^H-NMR (CDCl_3_) δ/ppm 7.51–7.49 (m, 2H), 7.38–7.31 (m, 3H), 3.08 (s, 1H); 1,4-Diphenylbuta-1,3-diyne data reported in literature [47]: ^1^H-NMR (270 MHz, CDCl_3_) δ/ppm 7.51–7.55 (m, 4H), 7.30–7.40 (m, 6H).

## 4. Conclusions

In this work, it is confirmed that C-H acidic terminal alkynes have relatively high reactivity in CuAAC reaction. It is also discovered that alkynes that are precursors of CuAAC-accelerating poly(triazolylmethyl) ligands have relatively high reactivity despite having a low acidity on par with the typical aliphatic terminal alkynes. Alkyne deprotonation and azide binding to copper are the slow steps in the CuAAC pathway, which considerably influence the rate of the overall reaction [41]. Consequently, it is understandable that acidic alkynes (i.e., **1**–**13**) and chelating azides such as 2-picolyl azide have high reactivity in CuAAC reactions. The fact that aliphatic alkynes **20** and **21** with relatively low C-H acidity are also fast-reacting is somewhat a myth. Indeed these compounds, or their triazole products from CuAAC, bear resemblance to the poly(triazolylmethyl) ligands such as TBTA that are known to accelerate CuAAC, yet it has not been entirely understood *how* TBTA accelerates the overall CuAAC reaction. It should also be noted that in this work the reactivity of these alkynes was compared in the presence of 2-picolyl azide, the most thoroughly studied chelating azide to date that delivers reliably high reactivity in CuAAC reactions. When the non-chelating benzyl azide was used in its place, the reactions were drastically deaccelerated, reaffirming that both azide and alkyne components have tremendous influence on the overall rate of the CuAAC reaction.

## Figures and Tables

**Scheme 4:**
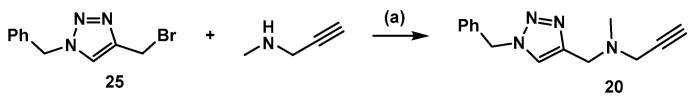
Synthesis of compound **20**. *Reagents and conditions*: (a) K_2_CO_3_, THF, r.t., 24 h, 60%.

**Scheme 5:**
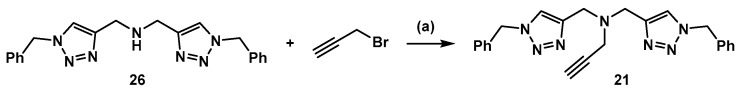
Synthesis of compound **21**. *Reagents and conditions*: (a) K_2_CO_3_, THF, rt, 24 h, 42%.

**Table 1:**
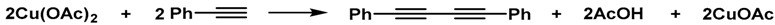
The effect of TBTA, TEA, and 2-picoline on oxidative homocoupling of phenylacetylene ^a^.

Entry	Additive	^1^H-NMR Yield ^b^
1	TBTA (0.02 mmol)	57%
2	None	0
3	2-picoline (0.02 mmol)	14%
4	TEA (0.02 mmol)	0

^a^ Conditions: phenylacetylene (0.2 mmol), Cu(OAc)_2_·H_2_O (0.2 mmol), *t*-BuOH (1 mL), r.t., air, 18 h; ^b^ Yields of 1,4-diphenylbuta-1,3-diyne were obtained from the ^1^H-NMR spectra of the reaction mixtures.
